# Toxic anthropogenic signature in Antarctic continental shelf and deep sea sediments

**DOI:** 10.1038/s41598-018-27375-4

**Published:** 2018-06-14

**Authors:** Enrique Isla, Elisabet Pérez-Albaladejo, Cinta Porte

**Affiliations:** 1 Institut de Ciències del Mar-CSIC, Passeig Marítim de la Barceloneta, 37-49. 08003 Barcelona, Spain; 2 Institut de Diagnosi Ambiental i Estudis de l’Aigua-CSIC, Jordi Girona 18-26, 08034 Barcelona, Spain

## Abstract

Industrial activity generates harmful substances which can travel via aerial or water currents thousands of kilometers away from the place they were used impacting the local biota where they deposit. The presence of harmful anthropogenic substances in the Antarctic is particularly surprising and striking due to its remoteness and the apparent geophysical isolation developed with the flows of the Antarctic Circumpolar current and the ring of westerly winds surrounding the continent. However, long-range atmospheric transport (LRAT) of pollutants has been detected in the Antarctic since the 70’s along the Antarctic trophic food web from phytoplankton to birds. Still, no information exists on the presence of cytotoxic compounds in marine sediments neither at basin scales (thousands of kilometers) nor in water depths (hundreds of meters) beyond shallow coastal areas near research stations. Our results showed for the first time that there is cytotoxic activity in marine sediment extracts from water depths >1000 m and along thousands of kilometers of Antarctic continental shelf, in some cases comparable to that observed in Mediterranean areas. Ongoing anthropogenic pressure appears as a serious threat to the sessile benthic communities, which have evolved in near isolation for millions of years in these environments.

## Introduction

The presence of harmful anthropogenic substances such as persistent organic pollutants (POPs) on our planet is ubiquitous^1–3^. POPs (e.g., polycyclic aromatic hydrocarbons (PAHs), polychlorobiphenyls (PCB), organochlorine pesticides) have been observed along the Antarctic trophic food web from phytoplankton to birds^4–6^. However, scarce information exists on the toxicity of marine sediment beyond the coastal fringes off Antarctic research stations^7–10^ perhaps because they were considered pristine environments, what inhibited investigations on this line. Benthic communities living there evolved in near isolation for millions of years under geophysical and thermal circumstances that shaped their present status, which resembles the benthic assemblages of the Cretaceous^11^. The modern Antarctic benthic assemblages show endemism of 50% to 60% at species level and are comparable to those found in tropical regions in terms of abundance and diversity^12,13^. The conditions favoring the retention of apparently archaic features in the Antarctic benthos remain unknown; however, these are unique communities and deserve special conservation measures due to their fragile evolutionary situation^11^. Based on the known persistence of anthropogenic pollutants in the environment^1^, here we present evidence of the presence of anthropogenic bioactive harmful substances in continental shelf and glacial trough sediments in the Weddell Sea and the vicinities of the Antarctic Peninsula (AP) on several spots along a coastline of approximately 4500 km. This is an ongoing serious threat to a large community of sessile organisms that can reach up to 100 g C m^−2^ and more than 17000 species^12,13^, which won’t have the chance to escape.

**Figure 1:**
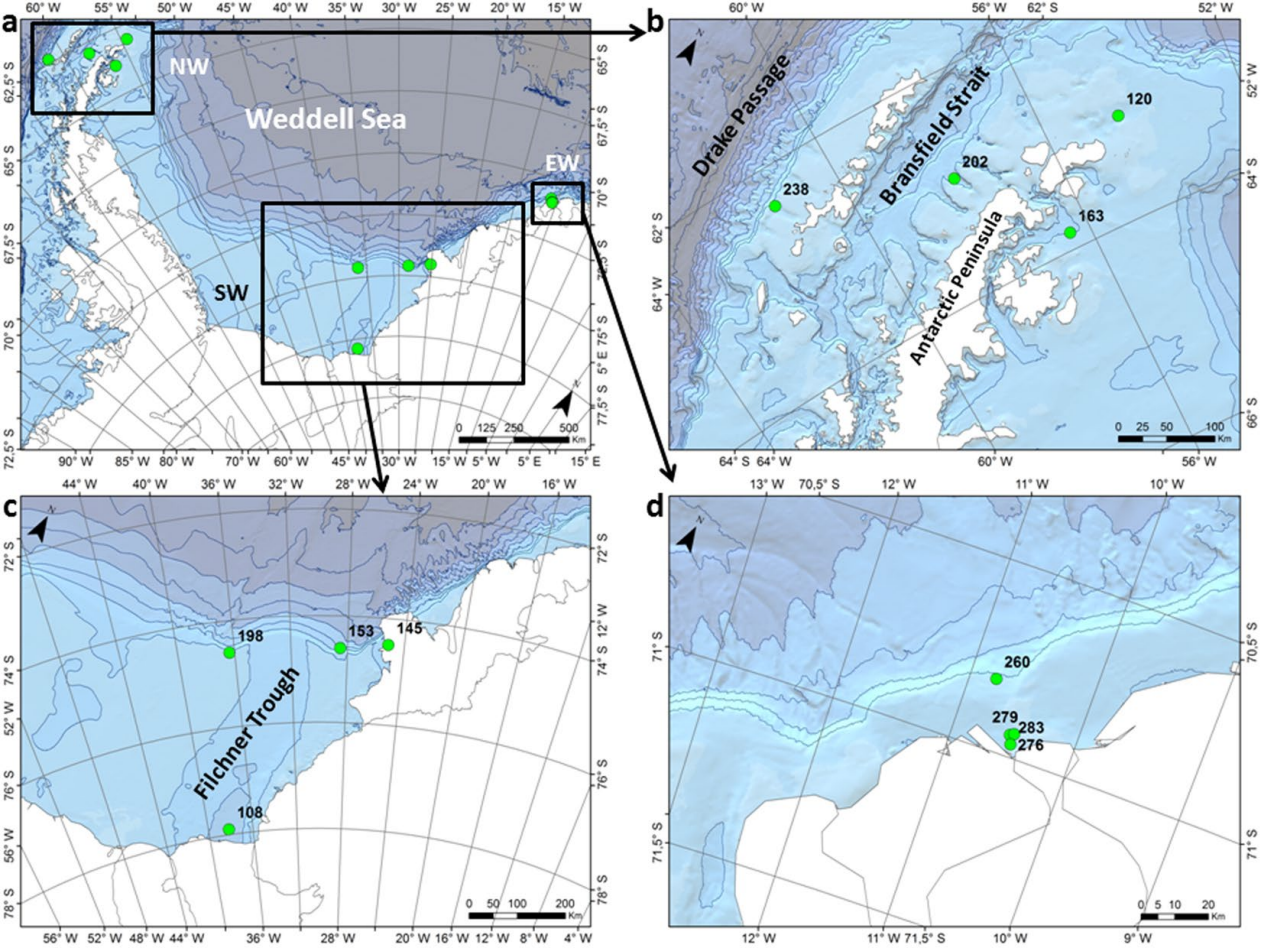
(**a**) General map of the Weddell Sea showing the study area, with magnified sections of the (**b**) northern (NW), (**c**) southern (SW) and (**d**) eastern Weddell Sea (EW). Black squares are not in scale with the map. This figure was created with the software ESRI ArcGIS ArcInfo version 10 and bathymetric information from the International Bathymetric Chart of the Southern Ocean^43^.

The extent of the study area enables the comparison of regions of the Antarctic with different exposure to anthropogenic impact (Fig. 1, Table 1). We assumed that the impact of long-range atmospheric transport (LRAT) of harmful substances is still rather low and uniform over the continent^1^, whereas in the vicinities of the AP where research stations and ship transit concentrates^14^, should be higher. The AP region hosts 33 of the 77 Antarctic research facilities and receives more than 95% of touristic landings, 98% of which reach the Antarctic by ship^15^. Thus, we anticipate a decreasing gradient in sediment toxicity (anthropogenic impact) from the AP region to the southern Weddell Sea off the Filchner Ice Shelf, where human access (both by ship and aircrafts) is rather limited and few research stations exist.

**Table 1:** Stations list.

Station number	Water depth (m)	Latitude	Longitude	Region	Sea floor relief characteristics
108	1216	77°54,17′S	38°09,99′W	SW	GT
145	702	74°49,80′S	25°07,44′W	SW	GT
153	1176	74°36,85′S	28°30,57′W	SW	GT
198	422	74°36,21′S	36°21,31′W	SW	CS
120	494	63°04.78′S	54°31.45′W	NW	CS
163	517	63°50.97′S	56°25.24′W	NW	CS
202	757	62°56.00′S	58°0.55′W	BS	GT
238	459	62°20.82′S	61°19.95′W	DP	CS
260	447	70°48,86′S	10°47,18′W	EW	CS
276	294	70°56,59′S	10°32,00′W	EW	CS
279	241	70°56,23′S	10°30,18′W	EW	CS
283	279	70°57,99′S	10°30,23′W	EW	CS

GT and CS stand for glacial trough and continental shelf, respectively. SW, NW, EW, BS and DP stand for southern Weddell Sea, northern Weddell Sea, eastern Weddell Sea, Bransfield Strait and Drake Passage, respectively.

An efficient approach to evaluate the influence of anthropogenic harmful substances in the environment is by assessing the cytotoxicity of pollutants and the effects of mixtures of contaminants on organisms^16–19^. To estimate the biological activity of unknown chemicals present in water and marine sediments, different successful *in vitro* bioassays have been developed integrating the potential additive, synergistic or antagonistic interactions among such chemicals and their bioavailability^17,20^. Interactions between pollutants and the biota initially take place at molecular and cellular levels. Therefore, the responses detected at these levels are considered the first manifestation of toxicity and their evaluation provides an effective assessment of the early impacts of chemical exposure^21^. These responses do not always imply effects at higher levels of organization, but they are a prerequisite for whole organism and population responses^22^. Based on the key role of the liver in the xenobiotic metabolism (detoxification), fish hepatocytes and permanent liver cell lines have been used as cellular *in vitro* models. A well-established permanent fish cell line is PLHC-1, derived from a topminnow (*Poeciliopsis lucida*) hepatocellular carcinoma. This cell line express the aryl hydrocarbon receptor (AhR) and the isoenzyme CYP1A after exposure to persistent harmful substances such as PAHs, PCBs, dioxins and pharmaceuticals as well as extracts of environmental matrices such as sediment^16,19^. CYP1A induction in fish hepatic cells (PLHC-1 and RTL-W1) has been associated with the presence of high molecular weight PAHs in marine sediments^23^, but other AhR ligands can induce the activity, as PCBs, furans, pesticides and dioxine-like compounds^16,22^. In cell-based bioassays EROD activity, a catalytic measure of CYP1A induction, facilitates the determination of dose-response relationships allowing the detection of CYP1A inducers^17^.

## Results and Discussion

**Figure 2:**
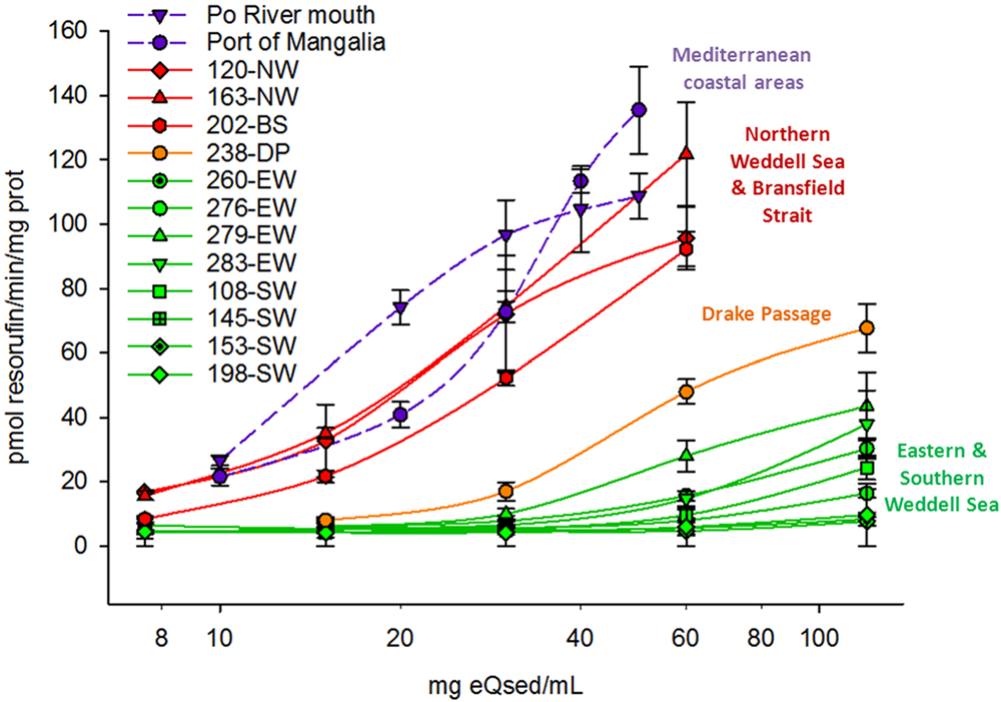
CYP1A induction in PLHC-1 cells exposed for 24 h to different concentrations of sediments extracts. Y axis indicates EROD activity as pmol of resorufin generated per minute and per mg of protein in exposed fish cells. Values are expressed as mean ± SEM of at least three independent assays. The positive control, 1 µM of BNF, lead to an EROD activity of 34.4 ± 0.7 pmol/min/mg protein. Stations 120 (blue line), 163 (red) and 202 (orange) show the highest ability to induce EROD activity in fish cells, followed by 238 (green), which indicates the presence of CYP1A agonists (e.g. high molecular weight PAHs, PCBs, etc.) in the extracts. Minor (e.g. 279) or no (e.g. 198) response is observed for the other sediment extracts, suggesting that those areas have less anthropogenic impact. For comparative purposes, purple dotted lines indicate the response of sediments from Mediterranean and Black sea coastal areas (e.g., the Po River mouth and the port of Mangalia)^19^.

**Figure 3:**
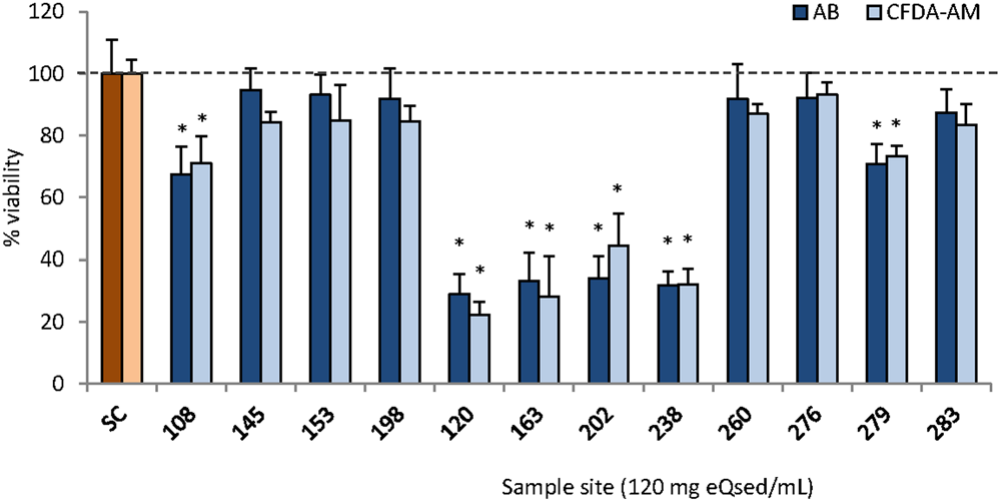
Cytotoxicity (AB and CFDA-AM) in fish cells PLHC-1 after 24 of exposure to sediment extracts at concentration of 120 mg eQsed · mL^−1^. Y axis shows the percentage of viability respect to no exposed cells. Values are expressed as mean ± SEM of at least three independent experiments. SC: Control Cells. Dotted line indicates 100% viability. *Significant differences with respect to control cells. Sediment extracts from stations 120, 163, 202 and 238 show the highest cytotoxicity in PLHC-1 cells fish cells.

The dose-response curves of EROD activity assays revealed that sediments from sampling stations in the northern Weddell Sea (NW) and the Bransfield Strait (BS) (sts. 163, 120 and 202) had the highest ability to induce EROD activity, in contrast some stations (e.g., sts. 198, 153) from the SW did not induce EROD activity at all (Fig. 2, Table 1). In marine sediment extracts from areas known to suffer from high anthropogenic impact, such as the Po and Danube river mouths, the presence of CYP1A inducers yielded to comparable CYP1A responses to those observed in samples from NW and BS (Fig. 2, Table 1). The sediment extracts from the AP region (NW, BS and Drake Passage stations) also showed the highest content of cytotoxic compounds decreasing cell viability between 20% and 44% (Fig. 3).

Our findings revealed that the presence of harmful substances may affect a coast line of at least 4500 km along the continental shelf of the Weddell Sea and the Antarctic Peninsula, including regions with few research stations and low ship traffic (e.g., SW and EW), suggesting that these substances reached the Antarctic by LRAT (Fig. 3). The sequential evaporation and deposition events along an increasing latitudinal gradient, described as a “grasshopper” mechanism, increase the concentration of POPs in high latitudes^1^. Because of their lipophilic nature, POPs can be carried by sinking particulate organic matter downward to the sea floor sediments^1,10^. LRAT drives semi-volatile organic pollutants to high latitudes with an estimated transport time of a week or less^3,24^. POPs have been detected in the high-latitude Antarctic 235 km from the coastline; although, with lower levels than those observed in the Arctic^25^. The atmospheric distribution of PAHs over the Scotia, Bellingshausen and NW Weddell Seas showed that LRAT is a conveyor of PAHs to the Southern Ocean but its contribution seems to be smaller than the input from both, land based and ship-borne research and touristic activity^26^. Most ship traffic to the Antarctic takes place in the AP region and it has grown exponentially during the last 30 years^14,27,28^. On the other hand, this comparatively small region hosts approximately 43% of the Antarctic research facilities (http://www.comnap.aq). The spatial coincidence of these factors and our results suggests that the higher anthropogenic activity in the vicinities of the AP is already impacting the deep sea sediment and producing regional differences within Antarctica.

Ships and research stations release pollutants to the environment mainly through their exhaust combustion channels and wastewater discharge^13,27,29,30^. Harmful substances may be released directly into the sea^13,29,31^; however, snow also efficiently incorporates atmospheric organic pollutants and works as a vehicle that transfers its contents to the seasonal snowpack, sea ice, glaciers and ice caps which are reservoirs of harmful substances on time scales ranging from days to millennia^3,6,32,33^. It has been suggested that ongoing global warming may increase the incorporation of POPs into the Antarctic water column with releases from soil and snow, making the AP region an area with the potential to become a net POPs sink in the coming decades^34^. Our results showed that this prediction could be already taking place. When organic pollutants enter ice-covered regions, they become protected from photolysis by low ambient light levels, and from microbial degradation by low temperatures enhancing their persistence in the environment^3^. Darkness and low temperatures (−2 °C to 2 °C) are almost constant characteristics of the seabed environment of the Antarctic continental shelf, including the present study area^35,36^. In contrast, organic contaminants catalyzed by solar radiation in Polar Regions can develop new types of harmful substances with the potential to produce a greater impact on the environment than their precursors^37^. It has been suggested that photolytic modification of sediment associated pollutants (e.g., PAHs and PCBs) may increase their toxicity and coupled with the development of the Antarctic ozone hole and the increased incidence of UV radiation, could significantly increase hazards to marine life^38^.

**Figure 4:**
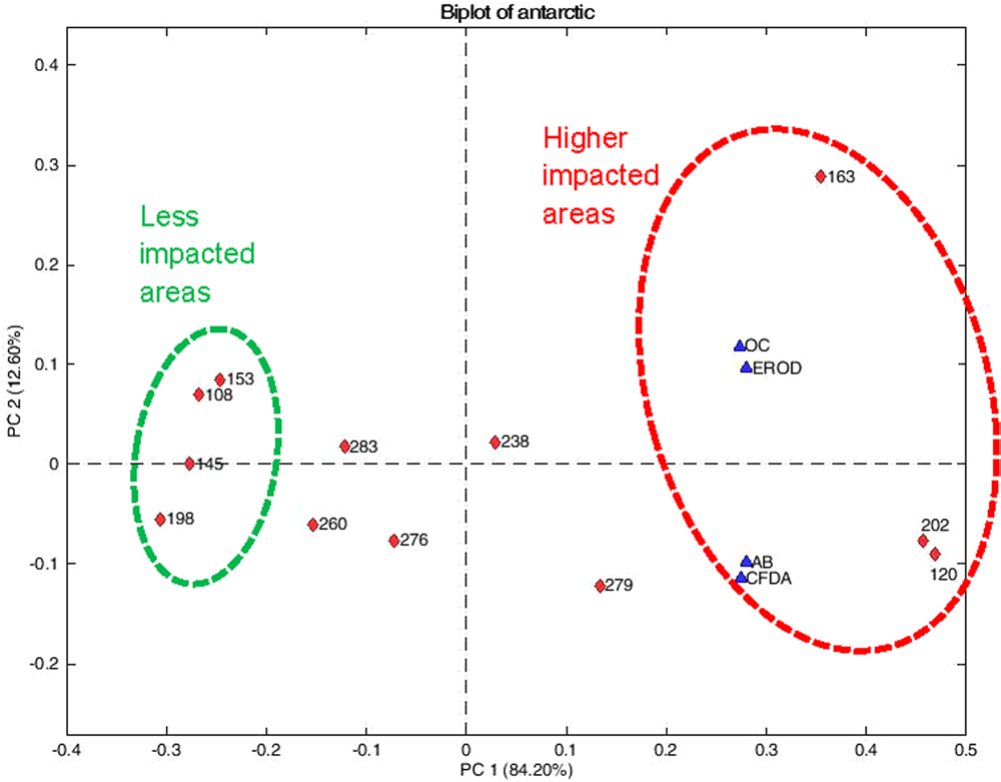
The principal component analysis (PCA) performed with two factors was able to explain 97% of the total variance, first factor (PC1) explaining 84.2%. PCA shows that organic carbon (OC) is closely related to CYP1A induction (EROD). Samples 163, 120 and 202 are on the more positive side of PC1, and had the highest concentration of OC and the highest ability to induce EROD activity, which means that they are the most impacted areas, followed by 279 and 238. The least impacted areas (198, 145, 108 and 153), are on the negative side of the PC1.

Toxicity found in samples collected far from coastal areas and in deep glacial troughs such as stations DP and BS, suggests that marine currents may transport harmful substances long distances (hundreds of kilometers) from coastal facilities, enhancing the spatial extent of such substances and adding to the LRAT input. Sediment resuspension is a submarine dispersal mechanism, which also represents a source of harmful substances^39^. Our findings revealed anthropogenic impacts on Antarctic deep-sea sediments, which will presumably increase if ongoing mechanisms (e.g., emission of pollutants to the atmosphere, ship traffic, research station releases) continue their “business as usual” trends. This situation is of great concern because there is no onsite remediation for the presence of harmful substances in deep sea deposits; only accumulation. Harmful substances of protracted persistence (e.g., PCBs, PAHs), may last longer in the Antarctic aided by low temperatures that lead to higher adsorption onto particles and lower degradation rates^2,3,32,33^. Adsorption onto biogenic particles enhances the export of PCBs and PAHs to the sediment column^40^. The association observed between organic carbon content and the facility to induce EROD activity (Fig. 4) suggests that the comparatively larger organic carbon fluxes in the region of the AP than in the EW^41,42^ facilitate the transport of harmful substances to the seabed. However, with the present data it is not possible to quantify the contribution of this process to the observed results. Certainly, sediment extracts could contain not only xenobiotics but natural organic substances presents in Antarctic continental shelf which may bind to AhR. Thus, a possible contribution of these organic substances to EROD induction cannot be discarded. However, based on our previous experience, this contribution is generally very low, which is supported by the low activity induced by sediment extracts collected in SW (e.g., sts. 198, 153).

Presumably, anthropogenic pollutants only begun to reach the Antarctic for the last century, whereas the Antarctic benthic fauna evolved in near isolation for millions of years and may not have time to cope with the serious threat that anthropogenic pollutants impinge even on the deep sea. This is the first report of the presence of CYP1A inducing agents and cytotoxic compounds in Antarctic sediments beyond the coastal fringe (>100 m water depth) and in high-latitude settings (78°S). Our observations are striking because they imply that harmful substances in the Antarctic are not restricted to the coastal areas near research stations or ship traffic pathways, but rather they persist and sink hundreds or even thousands of meters, until they reach the deeper ocean posing a serious threat to benthic communities distributed along wide bathymetric (approx. 1200 m water depth) and latitudinal (>15°) ranges.

## Methods

### Sediment sampling

12 stations were visited during R/V “Polarstern” expeditions PS77, PS81 and PS82 along the axis of glacial troughs and the continental shelves of the Weddell Sea, the Bransfield Strait and the Drake Passage (Fig. 1, Table 1). Sediment cores were collected with a multicorer that enables recovering samples with negligible losses of silt and clay and disturbance of the sediment-water interface (Barnett *et al*., 1984). The upper 5 mm of the sediment cores were subsampled in the laboratory of the ship and frozen at −20 °C on board. Back at the home Institution were freeze-dried before grinding in an agate mortar.

### Organic carbon

One gram of homogenized sediment underwent 6 M HCl vapor digestion overnight and set to dry for 48 h at 45 °C before combustion in a LECO TruSpec CN. Organic carbon analyses were run in triplicates and results expressed in % dry weight.

### Preparation for bioassays

Three grams of dried and homogenized sediment were extracted twice with 20 mL dichloromethane/hexane (1:1), followed by dichloromethane/acetone (1:1) in an ultrasonic bath^19^. The extracts were combined, evaporated, and reconstituted into 125 μL of dimethyl sulfoxide (DMSO). The stock was equivalent to 24 g dry weight extract (eQsed)·mL^−1^, and was serially diluted in DMSO to the desired concentrations.

### Cell culture

The PLHC-1 cell line (ATCC; CRL-2406), derived from topminnow (*Poeciliopsis lucida*) hepatocellular carcinoma, was routinely cultured in MEM supplemented with 5% FBS, 2 mM L-glutamine, 1 mM sodium pyruvate, 0.1 mM nonessential amino acids, 1.5 g·L^−1^ sodium bicarbonate, 50 U·mL^−1^ penicillin G and 50 μg·mL^−1^ streptomycin^19^. Application of sediment extracts to cell cultures was done by diluting the extracts in culture medium, which was then added to culture plate wells. The final concentration of DMSO in culture wells never exceeded 0.5% (*v/v*). For each assay, non-exposed controls were included, in them only the solvent (DMSO) was added to the culture medium.

### Cell viability

Two fluorescent dyes were used to assess cytotoxicity in PLHC-1 cells: Alamar Blue (AB), which estimates the metabolic activity, and 5-carboxyfluorescein diacetate acetoxymethyl ester (CFDA-AM), which monitor membrane impairment. PLHC-1 cells were seeded in 96-well plates at a density of 7.5 × 10^4^ cells per well (200 µL) and were allowed to attach for 24 h. After 24 h exposure of the cells to sediment extracts, the exposure medium was removed and 100 µL of a solution containing 5% AB and 4 µM CFDA-AM, was added to each well and the plate incubated for 1 h. Results were obtained as relative fluorescent units (RFUs) at the excitation/emission wavelengths pairs of 530/590 nm for AB, and 485/530 nm for CFDA-AM in a fluorescent plate reader (Varioskan, Thermo Electron Corporation). At least six replicates in three different plates were read to measure the cytotoxic effect of the extract at a given concentration by comparison to the fluorescence read in non-exposed cells. Metabolic impairment (AB) and disruption of membrane integrity (CFDA-AM) were determined in PLHC-1 cells after 24 h exposure to organic sediment extracts at 120 mg eQsed·mL^−1^.

### Induction of EROD activity

The CYP1A assay was performed as described in Pérez-Albaladejo *et al*.^19^. PLHC-1 cells were seeded at 6.5 × 10^5^ cells per well, in 48-well plates and allowed to grow for 24 h. Then, cells were exposed for 24 h to sediment extracts in a final volume of 250 µL. 1 μM of BNF and the solvent (0.5% DMSO) were used as positive control and blank, respectively. After exposure, cells were rinsed with PBS and incubated for 15 min, with 2 μM 7-ethoxyresorufin at 30 °C. The fluorescence was read in a microplate reader (Varioskan, Thermo Electron Corporation), at the excitation/emission wavelength pairs of 537/583 nm. The total cellular proteins were measured with fluorescamine using bovine serum albumin (BSA) as standard. EROD activity was quantified by calibration with 7-hydroxyresorufin. The results were expressed as pmol of resorufin formed per minute and per milligram of protein (pmol/min/mg protein).

### Statistical analyses

The concentration of sediment extract resulting in 50% effect (EC_50_) and the R_BNF_ were calculated by using Sigmaplot 11.0 software. Statistical differences were analyzed by one-way ANOVA with Dunnett test, using SPSS 19.0. Level of significance was set at *p*-value < 0.05. The principal component analysis (PCA) was carried out with PLS Toolbox 8.2/MatLab software.

### Data availability statement

All data generated or analyzed during this study are included in this published article.
